# Supplementary material for: Age-Related sncRNAs in Human Hippocampal Tissue Samples: Focusing on Deregulated miRNAs
Source: Int J Mol Sci. 2024 Nov 29;25(23):12872. doi: 10.3390/ijms252312872 (PMC11641170; doi:10.3390/ijms252312872)
Supplement: Supplementary file 1 [file ijms-25-12872-s001.zip › ijms-3276692-supplementary.pdf]

Supplementary tables

Table S1. DE miRNA obtained with the differential expression analysis

Old vs young comparison				
Transcript ID	Accession	Fold Change	p-value	Chromosome Location (First nucelotide)
hsa-miR-1825	MIMAT0006765	3,42	0,009	Chr20:30825633
hsa-miR-5571-5p	MIMAT0022257	2,40	0,016	Chr22:23228471
hsa-miR-4648	MIMAT0019710	2,15	0,032	Chr7:2566708
hsa-miR-5096	MIMAT0020603	-2,05	0,003	Chr4:79747918
hsa-miR-551b-3p	MIMAT0003233	-2,12	0,023	Chr3:168269702
hsa-miR-3175	MIMAT0015052	-2,14	0,005	Chr15:93447638
snR38A	snR38A	-2,26	0,002	Chr17:74557715
hsa-miR-7975	MIMAT0031178	-2,61	0,033	Chr19:55634593
hsa-miR-4730	MIMAT0019852	-2,68	0,016	Chr17:78393227
hsa-miR-7846-3p	MIMAT0030421	-2,89	0,006	Chr:1:12227067
Centenarian vs old comparison				
Transcript ID	Accession	Fold Change	p-value	Chromosome Location (First nucelotide)
hsa-miR-199a-3p	MIMAT0000232	3,90	0,022	Chr1:172113694
hsa-miR-199b-3p	MIMAT0004563	3,90	0,022	Chr9:131007024
hsa-miR-451a	MIMAT0001631	3,70	0,005	Chr17:27188421
hsa-miR-203a	MIMAT0000264	3,41	0,026	Chr14:104583806
hsa-miR-192-5p	MIMAT0000222	2,81	0,024	Chr11:64658675
hsa-miR-4314	MIMAT0016868	2,63	0,041	Chr17:27188421
hsa-miR-664b-3p	MIMAT0022272	2,24	0,034	ChrX:153996910
hsa-miR-3136-5p	MIMAT0015003	2,02	0,008	Chr3:69098155
ENSG00000239154	ENSG00000239154	-2,05	0,037	Chr11:101929036
hsa-miR-185-3p	MIMAT0004611	-2,16	0,021	Chr22 :20020711
hsa-miR-3135b	MIMAT0018985	-2,30	0,030	Chr6:32717729
Centenarian vs young comparison				
Transcript ID	Accession	Fold Change	p-value	Chromosome Location (First nucelotide)
hsa-miR-1825	MIMAT0006765	6,27	0,025	Chr20: 30825633
hsa-miR-5571-5p	MIMAT0022257	4,06	0,030	Chr22: 23228471
hsa-miR-4314	MIMAT0016868	3,44	0,015	Chr17: 7991384
hsa-miR-6826-5p	MIMAT0027552	3,17	0,008	Chr3: 128990994
hsa-miR-642b-3p	MIMAT0018444	3,03	0,037	Chr19: 46178199
hsa-miR-4442	MIMAT0018960	2,79	0,009	Chr3: 25706366
ENSG00000208308	ENSG00000208308	2,55	0,019	Chr2: 135894198
hsa-miR-4648	MIMAT0019710	2,40	0,013	Chr7: 2566708
hsa-miR-2276-3p	MIMAT0011775	2,39	0,041	Chr13: 24736608
hsa-miR-3935	MIMAT0018350	2,34	0,025	Chr16: 56279489
hsa-miR-6507-3p	MIMAT0025471	2,21	0,044	Chr10: 100684259
hsa-miR-519e-5p	MIMAT0002828	2,00	0,041	Chr19: 54183207
hsa-miR-4290	MIMAT0016921	2,00	0,017	Chr9: 92785733
hsa-miR-550a-3-5p	MIMAT0020925	-2,01	0,028	Chr7: 29720405

hsa-miR-3910	MIMAT0018184	-2,03	0,009	Chr9: 94398595
ENSG00000238816	ENSG00000238816	-2,09	0,002	Chr4: 79561254
hsa-miR-3175	MIMAT0015052	-2,14	0,018	Chr15: 93447638
hsa-miR-5096	MIMAT0020603	-2,51	0,010	Chr4: 79741918
hsa-miR-4478	MIMAT0019006	-2,62	0,047	Chr9: 124882426
hsa-miR-4730	MIMAT0019852	-3,32	0,040	Chr17: 78393227

Table S2. miRNA obtained with the Pearson correlation analysis

Positive correlation				
Transcript ID	Accession	r Pearson	p-value	Chromosome Location (First nucelotide)
hsa-mir-29b-2	MI0000107	0.80	0.0002	Chr1: 207975788
mgU6-53	mgU6-53	0.78	0.0004	Chr14: 21865452
hsa-miR-6826-5p	MIMAT0027552	0.78	0.0004	Chr3: 128990994
hsa-miR-4441	MIMAT0018959	0.77	0.0005	Chr2: 240007524
hsa-let-7b-3p	MIMAT0004482	0.77	0.0005	Chr22: 46509625
hsa-miR-642b-3p	MIMAT0018444	0.67	0.0042	Chr19: 46178199
hsa-miR-1825	MIMAT0006765	0.67	0.0044	Chr20: 30825633
hsa-miR-5571-5p	MIMAT0022257	0.67	0.0049	Chr22: 23228471
hsa-miR-6129	MIMAT0024613	0.66	0.0054	Chr17: 47365778
hsa-mir-195	MI0000489	0.64	0.0070	Chr17: 6920934
hsa-miR-3679-5p	MIMAT0018104	0.64	0.0074	Chr2: 134884701
hsa-miR-1273g-3p	MIMAT0022742	0.64	0.0077	Chr1: 53406042
hsa-miR-4442	MIMAT0018960	0.64	0.0082	Chr3: 25706366
hsa-mir-29a	MI0000087	0.63	0.0090	Chr7: 130561506
hsa-miR-197-5p	MIMAT0022691	0.63	0.0094	Chr1: 110141523
hsa-mir-885	MI0005560	0.63	0.0095	Chr3: 10436173
Negative correlation				
Transcript ID	Accession	r Pearson	p-value	Chromosome Location (First nucelotide)
ENSG00000221750	ENSG00000221750	-0.77	0.0005	ChrX: 54953738
hsa-miR-3175	MIMAT0015052	-0.72	0.0018	Chr15: 93447629
gi:555853	gi555853_copy0	-0.71	0.0020	
gi:555853	gi555853_copy1	-0.71	0.0020	
E3	E3	-0.70	0.0025	Chr3: 186505088
snR38A	snR38A	-0.70	0.0026	Chr17: 74557715
gi:555853	gi555853_copy2	-0.66	0.0050	
ENSG00000265651	ENSG00000265651	-0.66	0.0055	
U36C	U36C	-0.66	0.0055	Chr9: 136217701
ENSG00000202252	ENSG00000202252	-0.65	0.0063	Chr11: 122930043
ACA19	ACA19	-0.65	0.0067	Chr10: 120819523
ENSG00000238816	ENSG00000238816	-0.65	0.0068	Chr4: 79561254
ENSG00000252433	ENSG00000252433	-0.63	0.0083	Chr1: 67568328
70qll_7	70qll_7	_6¡❷9	6¡66❹❸	Chr70¿76707❷7❸6

.The.empty.cells.means.not.results.for.this.transcript.in.the.new.version.of.the.genome¡

Table S3. miRNA candidates selected for validate

miRNA	Type of analysis	Comparison
hsa-miR-6826-5p	Differential expression / Correlation	Centenarian vs young
hsa-let-7b-3p	Correlation	-
hsa-miR-4441	Correlation	-
hsa-miR-1825	Differential expression / Correlation	Old vs young<br>Centenarian vs young
hsa-miR-451a	Differential expression	Centenarian vs old
hsa-miR-7846	Differential expression	Old vs young

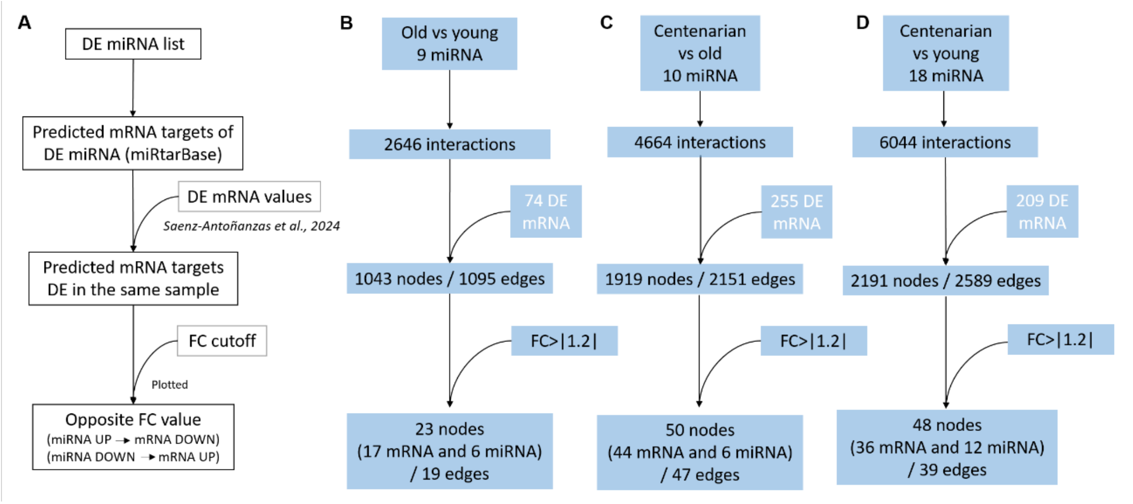

Figure S1. miRNA-mRNA networks workflow. (A) The Cytoscape networks were built following each of the represented steps considering the (B) old vs young, (C) centenarian vs old, and (D) centenarian vs young comparisons. DE: differentially expressed. UP: upregulated. DOWN: downregulated. FC: fold change.
